# Evaluation of sympathetic skin response for early diagnosis and follow-up of diabetic peripheral neuropathy in children

**DOI:** 10.1186/s12887-023-04323-4

**Published:** 2023-09-23

**Authors:** Mei Jin, Jing Liu, Kang Liu, Ziwei Zhao, Suzhen Sun

**Affiliations:** 1 The Children Hospital of Hebei Province, Shijiazhuang, Hebei 050000 China; 2 The Key Laboratory of Pediatric Epilepsy and Neurological Disorders of Hebei Province, Shijiazhuang, Hebei 050000 China

**Keywords:** Children, Type 1 diabetes mellitus, Peripheral neuropathy, Sympathetic skin response, Nerve conduction study

## Abstract

**Background:**

The morbidity of type 1 diabetes mellitus (T1DM) in children is increasing and diabetic peripheral neuropathy (DPN) is one of the main microvascular complications of T1DM. The aim of this study was to explore sympathetic skin response (SSR) characteristics in children with T1DM and analyze the value of early diagnosis and follow-up in T1DM complicated with DPN.

**Methods:**

Our prospective study enrolling 85 participants diagnosed with T1DM and 30 healthy controls (HCs) in the Children’s Hospital of Hebei Province from 2017 to 2020. Compared the outcomes of SSR and nerve conduction study (NCS) in T1DM, and evaluated the variations in SSR and NCS of different durations, as well as changes after six months of therapy.

**Results:**

SSR latency of T1DM group showed statistical difference as compared to HCs (*p* < 0.05). The SSR test was more sensitive than the NCS test in the early diagnosis of T1DM with DPN (*p* < 0.05). The abnormal rates of SSR and NCS in long duration of disease were higher than those in short duration of disease (*p* < 0.05). Among 65 participants with diabetic neuropathy, the onset latencies of SSR were shortened and the NCS were improved after treatment (*p* < 0.05).

**Conclusions:**

SSR could provide the accurate early diagnosis and follow-up of pediatric diabetic peripheral neuropathy.

## Introduction

Type 1 diabetes mellitus (T1DM) is the major type of pediatric diabetes, affecting about two million children and adolescents globally, the annual incidence estimates are 98,200 (128,900) new cases in the under 14 years old [1], and the new cases are increasing overtime [2]. Diabetic peripheral neuropathy (DPN) is one of the main complications of T1DM, and symmetric sensory-motor axonal DPN is the one with the highest prevalence [3]. Nerve conduction studies (NCS), are noninvasive and well-tolerated by children, used mainly to assess the large myelinated peripheral nerves, what’ more, NCS are the gold standard exam for diagnosing DPN [4]. However, many studies have shown that the metabolic by-products from having diabetes, particularly when it is poorly controlled, damage the small unmyelinated peripheral nerve function prior to the large myelinated nerve fibre [5]. Sympathetic skin response (SSR), as an objective electrophysiological marker to evaluate the autonomic unmyelinated nerve fibre function, has been used in the diagnosis and prognosis assessment of many diseases, such as pediatric Guillain-Barré syndrome [6], amyotrophic lateral sclerosis [7], multiple sclerosis [8], and adult diabetes mellitus [9], etc. The SSR study of pediatric diabetes mellitus is rare, therefore, the purpose of our study is to explore SSR characteristics in children living with T1DM and analyze the value of early diagnosis and follow-up in T1DM complicated with DPN.

## Methods

### Participants

We prospectively recruited T1DM participants who were admitted to endocrinology department between October 2017 to March 2020. Inclusion criteria for the T1DM group were as follows: (I) participants aged ≥ four years and ≤ 14 years; and (II) those who fulfilled the WHO diagnostic criteria of type 1 diabetes mellitus [10]: participants with classic symptoms of hyperglycemia (such as polydipsia, polyuria or weight loss) or hyperglycemic crisis (diabetic ketoacidosis), either fasting plasma glucose (FPG) ≥ 126 mg/dl (7.0 mmol/l) or a random plasma glucose ≥ 200 mg/dl (11.1 mmol/l) or 2-h 75 g oral glucose tolerance test (OGTT) ≥ 200 mg/dl (11.1 mmol/l) or HbA1c (A1C) ≥ 6.5%. Participants with other known causes of peripheral neuropathy, such as infection, traumatic, metabolic, autoimmune, toxic or inherited peripheral neuropathy, were excluded.

Other children (aged ≥ five years and ≤ 14 years) diagnosed with Tourette’s syndrome were selected as the healthy controls (HCs). All HCs had unremarkable cranial magnetic resonance imaging findings, were not taking any drugs with anti-cholinergic effects, and they did not present with clinical features of nerve system damage or any other autoimmune and oncological diseases.

Legal guardians of all participants provided written informed consent before inclusion. This study was approved by the Ethics Committee of the Children’s Hospital of Hebei Province (202222-59).

### Sympathetic skin response study (SSR)

**Fig. 1:**
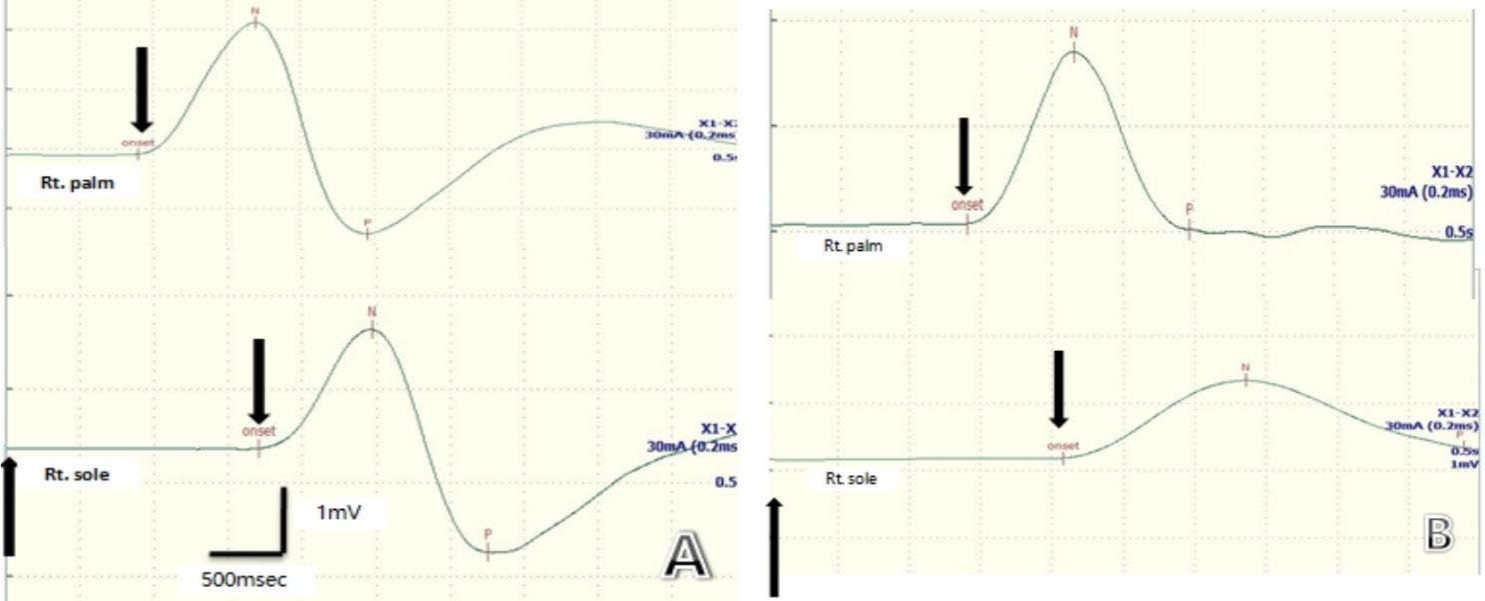
**A** The normal sympathetic skin response (SSR) were elicited from a 10-year-old boy. The upward pointing arrow indicated the electrical stimulus and the downward pointing arrow demonstrated the onset of the response. The SSR onset latency was measured from stimulus to onset of the response, N latency was measured from stimulus to the negative peak and P latency was measured from stimulus to the positive peak. **B** The abnormal sympathetic skin response (SSR) were elicited from a 11-year-old diabetic boy. The SSR latencies on the palm and sole were significantly prolonged. The onset latency of upper limb was 1.41s (97.50 percentile was 1.32s), the onset latency of lower limb was 2.10s (97.50 percentile was 1.80s)

All subjects lay comfortably on the bed in a quiet room. The temperature of the room and the limbs of the patient were maintained at 23–26 °C and 32.0–36.0 °C, respectively. We recorded the SSR of the upper and lower limbs using a electromyogram evoked potential system MEB2306C (Japan). Recording electrodes were placed on the palmar and plantar surfaces and reference electrodes were placed on the dorsum of the hands and feet. An electrical stimulus (intensity, 20–40 mA; duration, 0.2 ms) was applied to the contralateral median nerve at the wrist. To overcome habituation and the ensuing variability, a 40–60 s inter-stimulus interval was delivered randomly, and the intensity of the stimulation was increased progressively. At least five consecutive SSR potentials were recorded, and the mean of all the recorded SSR potentials was analyzed. SSR are made up of negative wave and positive wave and the parameters of SSR consist of the latency (onset, N and P latency) and the amplitude.The onset latency was measured from the beginning of the stimulus to the first continuous deflection from the baseline, N latency was measured from the stimulus to negative deflection, and P latency was measured from the stimulus to positive deflection, as shown in Fig. 1-A. Numerous studies have shown that the amplitude of SSR was highly variable and was an unreliable parameter due to the influence of habituation and age, whereas the latency was a stable parameter, free from repeated stimuli [11]. To avoid the influence of age on SSR, we age-matched 30 healthy controls with diabetic participants. The results were considered abnormal when no response was obtained or when the latency exceeded the 97.5th percentiles of HCs.

### Nerve conduction study (NCS)

All procedures were performed on the upper and lower limbs. The NCS study should include four motor nerves (peroneal, tibial,median and ulnar) and three sensory nerves (median, ulnar and sural). The parameters included motor conduction velocity (MCV), distal and proximal compound muscle action potentials (d/pCMAP), sensory conduction velocity (SCV) and sensory nerve action potential (SNAP). Results outside the 2.5 percentiles of values obtained in the age-matched healthy controls were considered anomaly.

### The course stratification of T1DM participants

Participants can be stratified after the definitive diagnosis of T1DM [12], the short duration of disease was usually less than three years, and the long duration was defined as three years or more after disease onset.

### The treatment of T1DM participants with peripheral neuropathy

Participants were defined as diabetic peripheral neuropathy when participants complained with the signs and/or symptoms of small and large fiber dysfunction or when the tests of NCS were abnormal. All participants with DPN received multiple insulin injections and oral vitamin B_12_ treatment for six months.

### Statistical analyses

Statistical analyses were performed using IBM SPSS Statistics version 24 (IBM Corp., Armonk, N.Y., USA). The distribution of all continuous variables was tested using the Kolmogorov–Smirnov normality test. Continuous variables with non-normal distributions were expressed as medians with interquartile ranges (IQR) and were compared using the Mann–Whitney U test. Categorical data of clinical features are shown as proportions and were compared using the chi-square test. Comparisons of SSR and NCS results between early diagnosis of T1DM were performed using McNemar’s tests. Statistical significance was set at 0.05.

## Results

### Baseline clinical features and SSR/ NCS findings in T1DM participants and HCs

A total of 85 participants with T1DM including 36 males and 49 females were recruited, with a median age of 10 years (IQR:8 ~ 12.5). The HCs were consisted of 30 participants, including 14 males and 16 females, with a median age of 11 years (IQR:8.8 ~ 12.3). There were no significant differences in terms of sex and age between T1DM and HCs (*p* > 0.05). Other clinical features of T1DM participants and HCs were summarized in Table 1.

In the T1DM group, 70 participants (82.4%) presented with classic diabetic symptoms (i.e. polydipsia, polyuria and weight loss) and 22 participants (25.9%) presented with diabetic ketoacidosis upon admission, of which five participants experienced onset symptoms of diabetic ketoacidosis. Twenty-four participants (28.2%) complained with the signs and/or symptoms of DPN.

The SSR latencies (including onset and N/P wave) of T1DM group in the upper and lower limbs were prolonged as compared to HCs (*p* < 0.05) (Fig. 1; Table 1). In addition, the results of NCS study (including sural, ulnar and median sensory nerve conduction as well as of peroneal and tibial motor nerve conduction) showed statistical differences when compared to the HCs (*p* < 0.05) (Table 1).

### Comparison of SSR and NCS in T1DM with DPN

There were 65 participants with diabetic peripheral neuropathy, the SSR test was more sensitive than the NCS test in the early diagnosis of T1DM with DPN (χ^2^=7.634, *p* = 0.006) (Table 2). Additionally, the abnormal rates of SSR and NCS in participants with a long duration of disease were higher than those with a short duration of disease (91.7% versus 56.2%, χ^2^=4.076, *p* = 0.043 for SSR and 75% versus 43.8%, χ^2^=4.009, *p* = 0.045 for NCS).

Among T1DM participants with DPN, 13.8% participants were the absent SSR in the upper and lower limbs simultaneously before treatment. The SSR latencies of upper and lower limbs were shortened after treatment (*p* < 0.05). Moreover, the parameters of NCS were also improved after treatment (*p* < 0.001) (Table 3).

**Table 1:** Comparison of clinical characteristics between T1DM and HCs

Variables	HCs	T1DM	Statistic values	*P-value*
Number	30	85		
Male/Female, n	14/16	36/49	*χ*^*2*^ = 0.168	0.682
Age,years,median(*IQR*)	11(8.8 ~ 12.3)	10(8 ~ 12.5)	*Z* = 0.440	0.660
Body Mass Index,Kg/m^2^,median(*IQR*)	16(15.4 ~ 19.9)	15.9(15.1 ~ 19.6)	*Z* = 0.679	0.497
Glycated hemoglobin, % median(*IQR*)	5.3(4.7 ~ 5.6)	9.4(8.9 ~ 13.2)	*Z* = 7.568	< 0.001
Duration of diabetes,years, median(*IQR*)		3(1 ~ 5)		
SSR (palm)				
Onset latency,sec, median(*IQR*)	1.21(1.15 ~ 1.24)	1.36(1.22 ~ 1.46)	*Z* = 4.792	< 0.001
N latency,sec, median(*IQR*)	1.85(1.78 ~ 1.92)	2.10(1.88 ~ 2.30)	*Z* = 4.334	< 0.001
P latency,sec, median(*IQR*)	2.88(2.73 ~ 2.96)	2.99(2.59 ~ 3.31)	*Z* = 1.973	0.049
SSR (sole)				
Onset latency,sec, median(*IQR*)	1.69(1.65 ~ 1.77)	1.77(1.61 ~ 1.99)	*Z* = 2.245	0.025
N latency,sec, median(*IQR*)	2.40(2.38 ~ 2.58)	2.58(2.35 ~ 2.92)	*Z* = 1.659	0.097
P latency,sec, median(*IQR*)	3.17(2.93 ~ 3.41)	3.49(3.14 ~ 3.95)	*Z* = 3.623	< 0.001
Median nerve,median(*IQR*)				
MCV (m/s)	54.5(53.2 ~ 57.0)	54.8(51.5 ~ 57.6)	*Z* = 0.392	0.695
CMAP (mV)	7.0(5.8 ~ 9.4)	7.3(5.8 ~ 9.2)	*Z* = 0.475	0.635
SCV (m/s)	55.2(52 ~ 56.5)	51.7(49.1 ~ 55.4)	*Z* = 2.950	0.003
SNAP (µV)	43.9(29.8 ~ 58.7)	36.5(23.9 ~ 49.7)	*Z* = 1.551	0.121
Ulnar nerve,median(*IQR*)				
MCV (m/s)	54.9(54.1 ~ 58.1)	54.7(52.8 ~ 57.8)	*Z* = 0.838	0.402
CMAP (mV)	5.9(4.4 ~ 6.7)	5.4(4.0 ~ 6.7)	*Z* = 0.602	0.547
SCV (m/s)	55.1(53.2 ~ 56.3)	51.5(47.3 ~ 54.2)	*Z* = 4.243	< 0.001
SNAP (µV)	22.4(15.9 ~ 35.6)	21.0(14.6 ~ 32.2)	*Z* = 0.803	0.422
Tibial nerve,median(*IQR*)				
MCV (m/s)	52.5(50.1 ~ 54.3)	46.3(44.7 ~ 48.4)	*Z* = 7.164	< 0.001
CMAP (mV)	9.7(8.9 ~ 10.9)	9.2(7.8 ~ 10.8)	*Z* = 1.564	0.118
Peroneal nerve,median(*IQR*)				
MCV (m/s)	50.5(49.6 ~ 53.5)	47.8(45.1 ~ 50.8)	*Z* = 3.988	< 0.001
CMAP (mV)	1.4(0.9 ~ 2.2)	1.3(0.8 ~ 2.0)	*Z* = 0.673	0.501
Sural nerve,median(*IQR*)				
SCV (m/s)	54.9(53.4 ~ 56.4)	49.0(45.6 ~ 55.6)	*Z* = 3.405	0.001
SNAP (µV)	5.7(2.8 ~ 7.8)	4.9(3.0 ~ 7.3)	*Z* = 0.838	0.402

Note. T1DM = Type 1 diabetes mellitus; HCs = Health controls ; SSR = Sympathetic skin response; MCV = Motor conduction velocity; CMAP = Compound muscle action potential;

SCV = Sensory conduction velocity; SNAP = Sensory nerve action potential; Z = Rank sum test.

**Table 2:** Comparison of SSR and NCS in the early diagnosis of T1DM with DPN, n(%)

		SSR	
		Positive	Negative
NCS	Positive	28(43.1%)	13(20%)
	Negative	24(36.9%)	0(0%)
*χ* ^*2*^		7.634	
McNemar, *P-value*		0.006	

Note. T1DM = Type 1 diabetes mellitus; SSR = Sympathetic skin response; NCS = Nerve conductive study

**Table 3:** Comparison of SSR and NCS before and after treatment in T1DM with DPN

Variables	Before treatment	After treatment	Statistic values	*P-value*
SSR (palm)				
Onset latency,sec, median(*IQR*)	1.40(1.26 ~ 1.49)	1.39(1.27 ~ 1.43)	*Z* = 2.130	0.033
SSR (sole)				
Onset latency,sec, median(*IQR*)	1.90(1.66 ~ 2.01)	1.80(1.74 ~ 1.90)	*Z* = 2.401	0.016
Median nerve SCV (m/s),median(*IQR*)	51.5(48.8 ~ 55.6)	55.3(51.6 ~ 56.9)	*Z* = 4.062	< 0.001
Ulnar nerve SCV (m/s),median(*IQR*)	50.6(47.2 ~ 54.2)	53.1(50.5 ~ 54.8)	*Z* = 4.268	< 0.001
Tibial nerve MCV (m/s),median(*IQR*)	45.7(44.3 ~ 47.7)	47.8(46.3 ~ 49.4)	*Z* = 5.585	< 0.001
Peroneal nerve MCV (m/s),median(*IQR*)	46.8(44.9 ~ 50.5)	49.4(47.7 ~ 52.8)	*Z* = 3.578	< 0.001
Sural nerve SCV (m/s),median(*IQR*)	47.5(45.2 ~ 54.7)	53.9(47.5 ~ 57.0)	*Z* = 3.846	< 0.001

Note. T1DM = Type 1 diabetes mellitus; SSR = Sympathetic skin response; NCS = Nerve conductive study; SCV = Sensory conduction velocity; MCV = Motor conduction velocity;

Z = Rank sum test

## Discussion

T1DM is the most common subtype of pediatric diabetes mellitus, the overall annual increase is estimated at around 3% [13].It is currently speculated that genetic and environmental factors play an important role in the pathogenesis of T1DM, and the latter factors include unreasonable eating habits, lack of physical activity, a polluted environment, etc. [14]. All of these factors complicate the management of diabetes and make it harder to maintain good metabolic control. The classic symptoms of T1DM include polydipsia, polyuria and weight loss. Polyuria, increased nocturia, even enuresis, are often the onset symptoms and the possibility of diabetes should be considered if these symptoms occur in older children. The prevalence of one of the common complications of diabetes, diabetic ketoacidosis, ranges from approximately 15–70% in developed countries and is more common at onset in children aged under two years [15]. Therefore children aged under two years often present with mental malaise, blurred consciousness or coma, prolonged and irregular breathing with smell of acetone, nausea, vomiting, abdominal pain, or shock. In the present study, 82.4% of children patients (70/85) showed typical symptoms, and 25.9% of (22/85) patients were admitted to hospital due to diabetic ketoacidosis, with 5.9% (5/85) displaying diabetic ketoacidosis as an onset symptom.

As a major complication of diabetes, the morbidity of DPN varies from 9 to 97% [16, 17], the common symptoms and signs include numbness, prickling or stabbing, burning or aching pain, unequivocally decreased or absent ankle reflexes. Compared to adults, children tend to experience more subclinical neuropathy (that is no signs or symptoms of DPN) in the early stage of the disease [18]. Subclinical DPN may be reversible in the early stage with intensive interventions such as lifestyle changes and medical therapy. However, the cumulative nerve impairments eventually become irreversible and clinical signs or symptoms occur overtime, which seriously affect the treatment effect as well as prognosis of the children. Therefore, early detection of subclinical DPN in children may allow earlier intervention, which may reduce or delay the morbidity of DPN in adult life.

Nerve conduction study (NCS) are mainly used to assess the large myelinated motor and sensory nerves of limbs in the clinical environment [19], and are simple, non-invasive and well-tolerated tests to achieve early diagnosis and screening of DPN in children and adults [20–22]. In the present study, we found that slowing of motor nerve conductive velocity mainly occurred in the lower extremities and the slowing of sensory nerve conductive velocity occurred in the upper and lower limbs. The amplitudes of the upper and lower limbs in children living with diabetes showed no significant differences as compared to control subjects, suggesting that demyelination of large somatic nerve fibers were the main pathological change of peripheral neuropathy. Nerve fibers of the lower limbs in children living with diabetes were more frequently impaired than those fibers of upper limbs, with abnormal rates of NCS being 63.1%, which were earlier than the clinical manifestations of peripheral nervous impairment in T1DM.

Autonomic dysfunction is an important factor of morbidity and mortality among children living with diabetes [23, 24], the common evaluations include heart rate, blood pressure, sweating abnormalities, as well as the callus. However, some subjective questionnaires do not reflect any differences between the patients and healthy controls, whereas some objective function tests have not been used in children, owing to higher demand for cooperation or lack of specialized equipment [25, 26]. Autonomic neuropathy in children with T1DM is likely underestimated due to suboptimal screening and subclinical neuropathy [27]. Therefore, many studies have shown that the SSR to be a repeatable, non-invasive examination to evaluate autonomic function in limbs [28]. The high abnormality of SSR in adult patients with diabetic neuropathy has been highlighted by many authors [29, 30]. In this study, we found that latencies of SSR were prolonged or absent in the upper and lower limbs. The abnormal rates of SSR in DPN were up to 80%, which especially occurred in the lower extremities, an earlier clinical or subclinical manifestation of autonomic nervous impairment in T1DM. Meanwhile, the SSR tests were superior to the NCS tests, therefore, the SSR tests could provide early accurate diagnosis of DPN.

There are many recommended guidelines at present, some experts advocate commencing screening five years after the definitive diagnosis of T1DM in children [31], while some experts advocate commencing screening two or three years after disease onset due to the rising incidence and complications of children living with diabetes [12]. Therefore, SSR and NCS could play a prominent role in screening for the development of DPN. In this study, we found that the abnormal rates of SSR and NCS in the long duration of illness were higher than those in the short duration of illness, suggesting that pediatric DPN was related to the duration of disease, which has been confirmed in most adult living with diabetes [32, 33]. Meanwhile, 65 patients with DPN who received intensive intervention for six months showed a shortening of onset latencies of SSR and nerve conduction velocities were improved. This further proved the importance and necessity of SSR and NCS detection in children living with diabetes.

This study had some limitations. Firstly, most enrolled pediatric diabetic participants showed no signs or symptoms of peripheral neurology. We did not conduct cohort studies to determine whether patients with subclinical peripheral neurology would exhibit obvious clinical manifestations in future. Secondly, our study did not conduct other autonomic function tests used in children living with diabetes.

In conclusion, SSR could evaluate the small unmyelinated sympathetic fibers and NCS could assess the large myelinated peripheral nerves, as they were both generally well-tolerated by children. The SSR tests were superior to the NCS tests in the early diagnosis of neuropathy, therefore, SSR could provide the accurate early diagnosis as well as follow-up of neuropathy.

## Data Availability

The datasets generated and/or analysed during the current study are not publicly available due to the data protection and privacy of the patients hospitalized at the Children’s Hospital of Hebei province, but are available from the corresponding author on reasonable request.
